# Can the body slope of interference screw affect initial stability of reconstructed anterior cruciate ligament?: An in-vitro investigation

**DOI:** 10.1186/s12891-021-04446-8

**Published:** 2021-06-18

**Authors:** Nazanin Daneshvarhashjin, Mahmoud Chizari, Javad Mortazavi, Gholamreza Rouhi

**Affiliations:** 1 Faculty of Biomedical Engineering, Amirkabir University of Technology, Tehran, Iran; 2 Institute for Orthopaedic Research and Training, Department of Biomedical science, Faculty of Medicine, Katholieke Universiteit Leuven, Leuven, Belgium; 3 Faculty of Mechanical Engineering, Sharif University of Technology, Tehran, Iran; 4 School of Physics, Engineering and Computer Sciences, University of Hertfordshire, Hatfield, UK; 5 Joint Reconstruction Research Center, Tehran University of Medical Sciences, Tehran, Iran

**Keywords:** ACL Injury and Reconstruction, Initial Stability, Bio-mimicked Interference Screw, Body Slope, Graft Damage, Graft Laxity, In-vitro Mechanical Tests

## Abstract

**Background:**

Superior biomechanical performance of tapered interference screws, compared with non-tapered screws, with reference to the anterior cruciate ligament (ACL) reconstruction process, has been reported in the literature. However, the effect of tapered interference screw’s body slope on the initial stability of ACL is poorly understood. Thus, the main goal of this study was to investigate the effect of the interference screw’s body slope on the initial stability of the reconstructed ACL.

**Methods:**

Based on the best screw-bone tunnel diameter ratios in non-tapered screws, two different tapered interference screws were designed and fabricated. The diameters of both screws were equal to bone tunnel diameter in one-third of their length from screw tip, then they were gradually increased by 1mm, in the lower slope (LSTIS), and 2 mm, in the higher slope (HSTIS) screws. To simulate the ACL reconstruction, sixteen soft tissue grafts were fixed, using HSTIS and LSTIS, in synthetic bone blocks. Through applying sub-failure cyclic incremental tensile load, graft-bone-screw construct’s stiffness and graft laxity in each cycle, also through applying subsequent step of loading graft to the failure, maximum load to failure, and graft’s mode of failure were determined. Accordingly, the performance of the fabricated interference screws was compared with each other.

**Results:**

HSTIS provides a greater graft-bone-screw construct stiffness, and a lower graft laxity, compared to LSTIS. Moreover, transverse rupture of graft fibers for LSTIS, and necking of graft in the HSTIS group were the major types of grafts’ failure.

**Conclusions:**

HSTIS better replicates the intact ACL’s behavior, compared to LSTIS, by causing less damage in graft’s fibers; reducing graft laxity; and increasing fixation stability. Nonetheless, finding the optimal slope remains as an unknown and can be the subject of future studies.

## Background

Anterior cruciate ligament (ACL) reconstruction is one of the most common orthopedic surgical procedures [1], in which hamstring tendon auto-graft is frequently used to reconstruct the ruptured ACL [2]. For the case of hamstring graft fixation, interference screw has become a popular choice, and many biomechanical studies reported equivalent or greater stability than other methods of fixation [3–5]. A wide variety of interference screw body geometries, for instance, cylindrical, tapered and hybrid were patented [6–11], which are designed to provide acceptable stability by creating squeezing pressure that holds tendon grafts into contact with the bone tunnel [12]. The interference screw needs to provide sufficient strength and stiffness, which are necessary for rehabilitation and daily activities before biological fixation is fully occurred [13].

Despite all advancement made regarding the ACL reconstruction, fixation of the graft in tibial bone tunnel, in the immediate postoperative period, due to poor bone mineral density, also because of the direction of applied load, which is mostly in the direction of the bone tunnel, is still a serious challenge [14–16]. Ten to twenty five % of patients still suffer from graft failure in the initial stage of rehabilitation [17], and in 9–22 % of reconstructions, a clinically important increase in anterior knee laxity was reported [18]. Moreover, the observed slippage of the graft might be attributed to the micro-motion between the graft and the interference screw within the bone tunnel under cyclic loading, which would eventually lead to loosening of the graft [19]. Graft irritation and laceration caused by metal interference screws could be another reason for some clinical failures [19, 20]. Furthermore, even a well-functioning ACL can be at the risk of traumatic rupture with a pooled rate of 5.8 %, at a minimum of 5-year follow-up [21].

Some previous *in-vitro* biomechanical studies investigated the effect of interference screw’s length, diameter, material properties, and different manufacturer designs on critical clinical outcomes (such as displacement and strength of the fixed graft), as well as its mode of failure, through applying cycling loading, and loading grafts to failure [3, 22–28]. Investigations on the effect of bone tunnel-interference screw diameter ratio implied that the use of a small diameter screw may cause graft slippage from the bone tunnel, and a larger screw diameter may damage the graft [12]. Morris et al. concluded that the screw’s diameter equal to, or 1 mm smaller than the tunnel’s diameter provides better fixation than using a screw with a 1 mm larger diameter, in which damages on the graft may occur [24]. On the other hand, Micucci et al. found that screw diameters ranging from 1mm less- to 2 mm greater than bone tunnel diameter can provide more satisfactory fixations [3]. Mann et al. found that fixation of tapered screws in tapered bone tunnel provides greater resistance to interference failure, compared with a non-tapered screw in the cylindrical bone tunnel, when the clearness between screw bone tunnel was equal in two groups [29]. Considering all screw designs’ variabilities, an important aspect of ACL reconstruction is to learn from, and mimic the intact insertion site of the ACL into tibial bone, which consists of four zones as follows: parallel fibers ligament; non-mineralized fibrocartilage; mineralized fibrocartilage; and bone [30]. At the insertion site, the gradient in material properties of the ACL allows effective load transfer, and thus minimizes stress concentration, and consequently reduces damage [30]. For this reason, in reconstruction procedure, by using a constant screw-bone tunnel ratio, the natural connection of the ACL into tibial bone cannot be followed. Considering the acceptable range of screw-bone tunnel diameter ratios (1mm less- to 2 mm greater than bone tunnel) [24] and the better performance of the tapered screws in comparison to the non-tapered ones [29], there is a need for a biomechanical comparison between the performances of different possible body slopes of the tapered interference screws, designed based on these acceptable range of screw-bone tunnel diameter ratios.

The main objective of this study was to investigate the effects of gradual increase of the diameter of tapered interference screws from equal diameter with the bone tunnel, known as the best screw-bone tunnel ratio, in one-third of their length from screw tip, where the engagement of screw and bone starts, to a 2 mm larger-, compared to a 1 mm larger than the diameter of the bone tunnel, on the stability of the reconstructed ACL fixation. It was hypothesized that by fixation of the interference screw with the higher body slope, compared to the lower one, intact ACL’s attachment to the bone tunnel can be better replicated, and thus it can provide a more stable graft fixation.

## Materials and methods

### Design and fabrication of the HSTIS and LSTIS

**Fig. 1:**
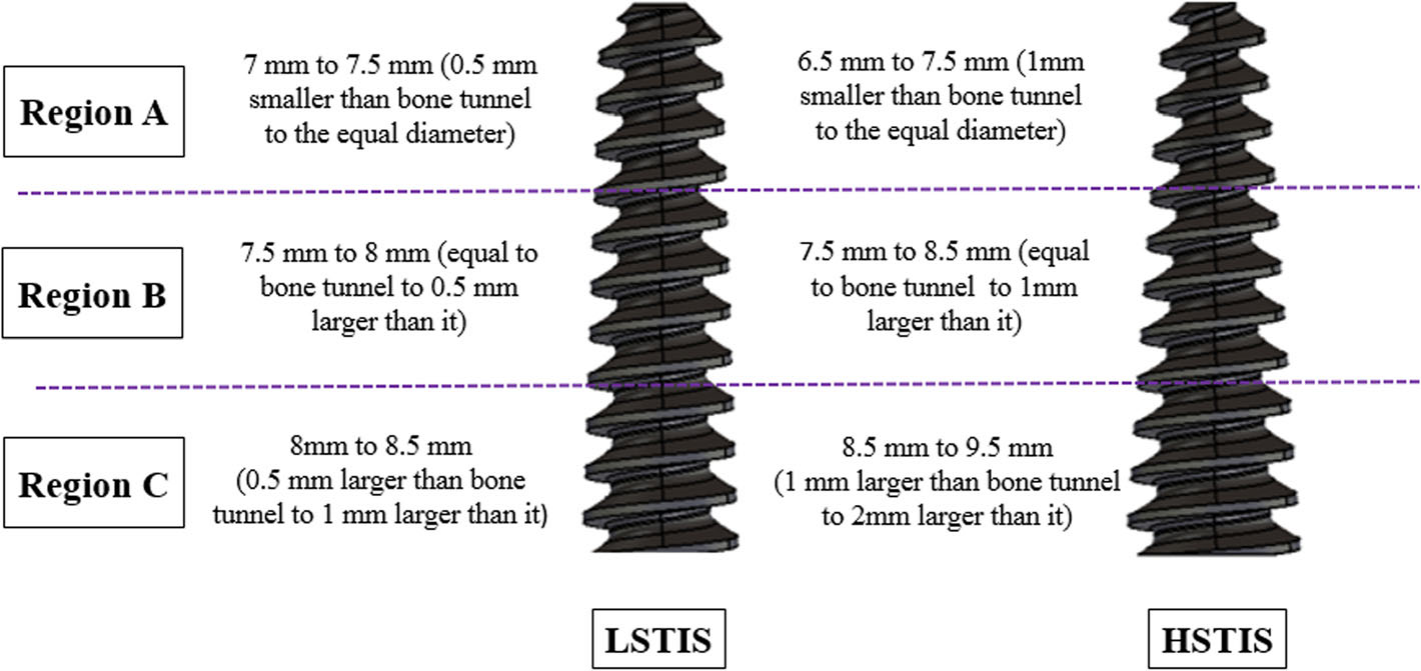
Changes in the diameters of two tapered interference screws: (Left) Lower slope tapered screw (LSTIS), and (Right) Higher slope tapered screw (HSTIS). Through regions B and C, the diameters of LSTIS and HSTIS were increased from 7.5 mm to 8.5, and from 7.5 to 9.5 mm, i.e. 1 and 2 mm greater than bone tunnel, respectively

Using a CNC TraubTx8 machine, prototypes of the two designed interference screws were made of CK45 steel, with a 30 mm length, a tapered body, and a flat head (Fig. 1). The screws had the same thread shape, with a 2.5 mm pitch, and 1.5 mm depth. The main difference between the designs of the two groups was the body slope of the screws. The diameter of both screws in one-third of their length, from the tip of the screw, was equal to bone tunnel diameter, which was gradually increased to 8.5 mm, and 9.5 mm, i.e. 1 and 2 mm larger than bone tunnel diameter. Accordingly, they were named lower slope tapered interference screw (LSTIS), and higher slope tapered interference screw (HSTIS) (Fig. 1). Therefore, in the LSTIS group, the average of screw diameter in one-third of its length from the tip (Fig. 1, region A) was larger, and in the rest of its length (Fig. 1, regions B and C) was smaller, compared to HSTIS group.

### In-vitro tests: comparison between LSTIS and HSTIS responses

Sixteen fresh bovine extensor tendons were cleared of adherent muscle fibers and surrounding soft tissues, wrapped, and stored frozen at -20 °C in sealed plastic bags for 3 weeks, in order to be used as soft tissue grafts [31]. On the day of testing, the tendons were thawed to room temperature (for 2–4 h), and all of them were kept moist with an 0.9 % normal saline solution during the sample preparation and test procedure [30]. Open-ended bone tunnels with a diameter of 7.5 mm were also created in the rigid polyurethane foam blocks (Sawbones, Pacific Research Laboratories, Inc., WA), with a density of 320 kg/m^3^ to simulate dense cancellous bone of tibial bone tunnel [32]. After preparation of the soft tissue and bone tunnel samples, looped bovine extensor digitorum tendon strands with a total length of 80 mm, were sized to 7.5 mm circumferentially by use of an ACL graft–sizing block.

**Fig. 2:**
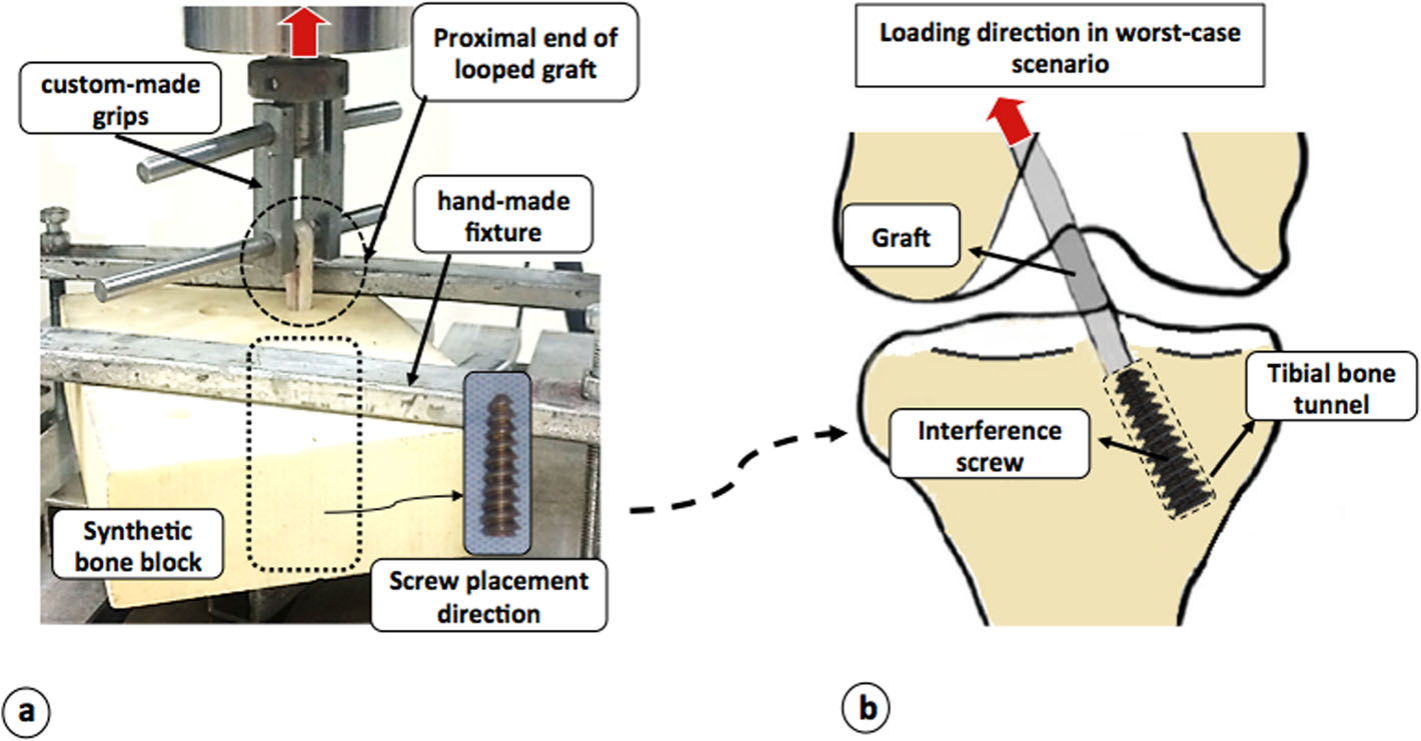
Experimental test set-up: **a** A looped graft captured in a custom-made rigs and fixed with an interference screw within a bone block, secured with a hand-made fixture, in the mechanical testing set-up; and **b** Position of tibial bone tunnel, direction of interference screw, and loading direction in the worst-case scenario in human body

**Fig. 3:**
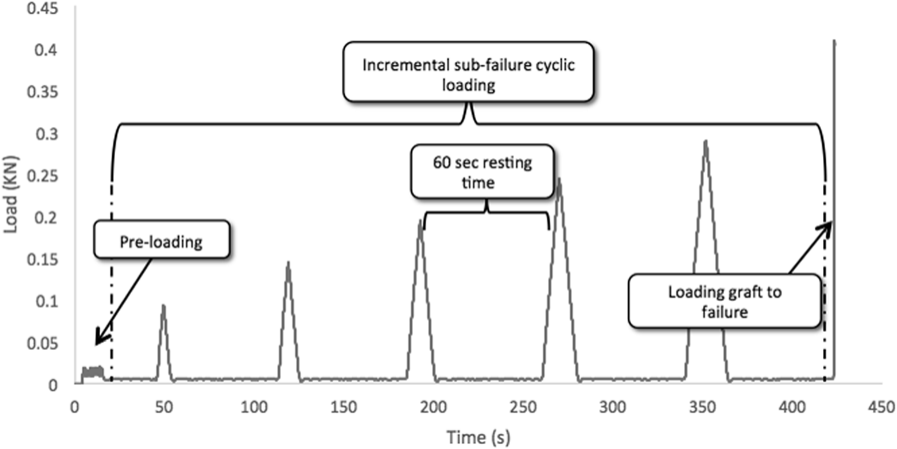
Loading steps in the current study: Pre-loading: sinusoidal tensile load changed from 5 to 20 N, with a frequency of 1 Hz; Incremental sub-failure cyclic loading: tensile load, with a rate of 25 N/sec to a peak value of 100, 150, 200, 250 and 300 N, then unloading and leaving the graft to be in rest for 60 s after each loading cycle; and finally loading graft to failure with a rate of 20 mm/s

Grafts were inserted in prepared bone tunnels in two groups. Then, HSTIS and LSTIS were placed concentrically between graft strands in the direction of the bone tunnel. Three centimeters of the proximal end of the looped tendon strands were kept free outside of the bone tunnel. The looped strands then were secured in the custom-made rigs in Zwick/Roell (Amsler HCT 25–400), and bone blocks were also fixed with a custom-made fixture (Fig. 2a). Immediately after the preparation of each graft-bone-interference screw sample, mechanical tests were carried out. By keeping graft strands perpendicular to the synthetic bone surface, the loading was applied parallel to the longitudinal axis of the tunnel (Fig. 2a). This boundary condition imitates the knee extension when the force vector of the ACL is in line with the tibial tunnel, which places maximal forces on a tibial graft fixation [3] (Fig. 2b). Various loading steps were applied to the grafts. First, a pre-loading, sinusoidal tensile load, ranging from 5 to 20 N, with a frequency of 1 Hz, for 10 cycles was applied to the graft. The preconditioning load ensures all specimens began the test under similar stress-strain conditions [22]. Then, an incremental sub-failure cyclic loading tensile load was applied. In this step, the graft was loaded with a rate of 25 N/sec to a peak value of 100, 150, 200, 250, and 300 N, then unloaded and left to be at rest for 60 s after each loading cycle [23]. An incremental loading and unloading allow measuring the amount of fixation stability, and graft laxity in different load levels, representing an aggressive, but typical rehabilitation loading levels [22]. Furthermore, for evaluating the reaction of the constructs under a sudden over-load event and maximum load capacity of the reconstructed graft, the graft was then loaded to failure immediately in tensile direction with a rate of 20 mm/s [33] (Fig. 3).

In each cycle of incremental sub-failure loading, the slope of the best-fit line to load-displacement curve of the loading phase was measured, as the bone-screw-graft’s stiffness, independent of the other cycles [22]. The construct stiffness in each load level represents the behavior of the structure in terms of fixation stability in the loading phase. Any difference, in the measured stiffness, between the two groups can be related to several reasons: (1) possible reversible elongation of the graft in the fixation site, when the applied force overcomes squeezing pressure caused by interference screw; or/and (2) slippage of the graft from the bone tunnel; or/and (3) graft fiber damages. Furthermore, in each sub-failure cycle after loading and unloading, to quantify irreversible changes in the graft, i.e. graft’s fiber damages and graft slippage from the bone tunnel, two other parameters were also measured: (1) The energy loss parameter, i.e. the area of the hysteresis curve during loading and unloading which indicates the difference between loading and unloading behavior of the construct (a large energy loss, which is associated with a sudden drop in reconstructed ACL force during unloading, is of clinical importance, as it may result in increased non-physiological loading of the knee joint, making it prone to early degeneration [22]); and (2) The graft laxity increase parameter, which measures the difference between the position of the graft before loading in each cycle and its position after the resting time, followed by loading and unloading in each cycle [23]. The graft slippage is also clinically important as it may increase working length of the graft in the joint, and subsequently laxity in the knee joint and loss of isometricity, which can create shear forces at the bone tunnel that prevents graft-bone integration [23]. In loading the graft to failure (step c), the bone-graft-screw’s stiffness; maximum load to failure; total displacement of the graft; and mode of graft failure were all recorded. The total displacement of the graft was defined as the difference between the initial position of the graft after pre-loading and its position at the failure point, along the longitudinal axis of the tunnel. Stiffness and maximum load to failure represent the stability of the constructs in sudden overloads. Moreover, total graft displacement measures accumulative graft slippage and displacement of the graft in sudden overload.

Statistical analysis was conducted with GraphPad Prism software (GraphPad Software, Inc.), version 6. In all groups, nonparametric distribution of the data was found using the Kolmogorow-Smirnow test [22]. Parameters of interest were statistically compared between the two groups using the Mann-Whitney U Wilcoxon rank-sum test [22]. In incremental sub-failure cyclic loading, the two groups were compared in each loading level, independent of the other load levels.

## Results

In the HSTIS group, by increasing the peak values of the load in incremental sub-failure cyclic loading to 200 N, 250 N, and 300 N, one, two, and three specimens’ fixation failed, respectively. On the other hand, fixation failure occurred for one, three and five specimens, in cycles with peak values of 150 N, 200 N, and 250 N, respectively, in the LSTIS group. Moreover, due to technical errors, one of the grafts, out of 8 grafts, failed in the HSTIS fixation procedure, so the number of total graft samples was seven and eight, for HSTIS and LSTIS groups, respectively.

**Fig. 4:**
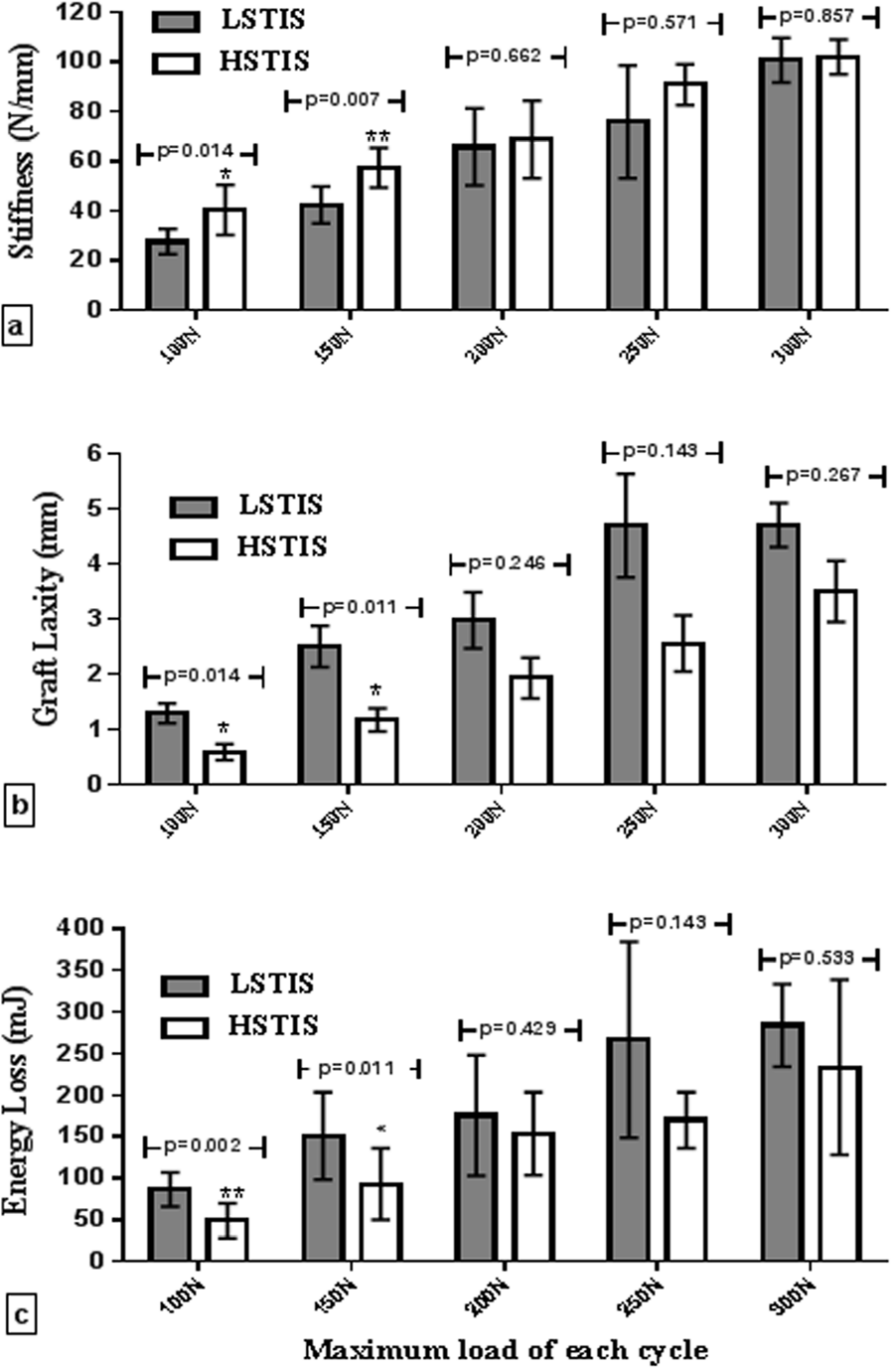
In-vitro tests results in different tensile sub-failure cycles: **a** Stiffness of the graft-bone-screw construct (N/mm); **b** Graft laxity parameter (mm); and **c** Energy loss (mJ) of low slope tapered interference screw (LSTIS) and high slope tapered interference screw (HSTIS) in different tensile sub-failure cycles, with a peak load value of 100, 150, 200, 250, and 300 N

An increase in the stiffness of the graft-bone-screw construct was observed in successive cyclic loading in each group (Fig. 4a). At the cycles with load peak values of 100 and 150 N, the stiffness of the graft-bone-screw construct in HSTIS vs. LSTIS was significantly different as follows: 40.73 ± 10.7 N/mm vs. 27.82 ± 5.10 N/mm (*P* < 0.05); and 57.70 ± 8.04 N/mm vs. 42.71 ± 7.51 N/mm (*P* < 0.01), respectively (Fig. 4a). Moreover, graft laxity increase parameter for the LSTIS group in all sub-failure cyclic load levels was greater than that of the HSTIS group (Fig. 4b). The graft laxity in LSTIS vs. HSTIS, showed a significant difference in cycles with load peak values of 100 and 150 N, i.e. 1.29 ± 0.51mm vs. 0.58 ± 0.38mm (*P* < 0.05); and 2.49 ± 0.99 mm vs. 1.17 ± 0.56 mm (*P* < 0.05), respectively. Regarding energy loss, in all sub-failure cyclic load levels, more energy was dissipated in LSTIS group, compared to the HSTIS group (Fig. 4c). A significantly greater amount of energy was dissipated in the LSTIS group, compared with the HSTIS group, at the cycles with load peak values of 100 and 150 N, i.e. 86.46 ± 20.23 mJ vs. 49.12 ± 21.03 mJ (*P* < 0.01); and 151.00 ± 52.46 mJ vs. 93.04 ± 43.25 mJ (*P* < 0.05), respectively.

**Fig. 5:**
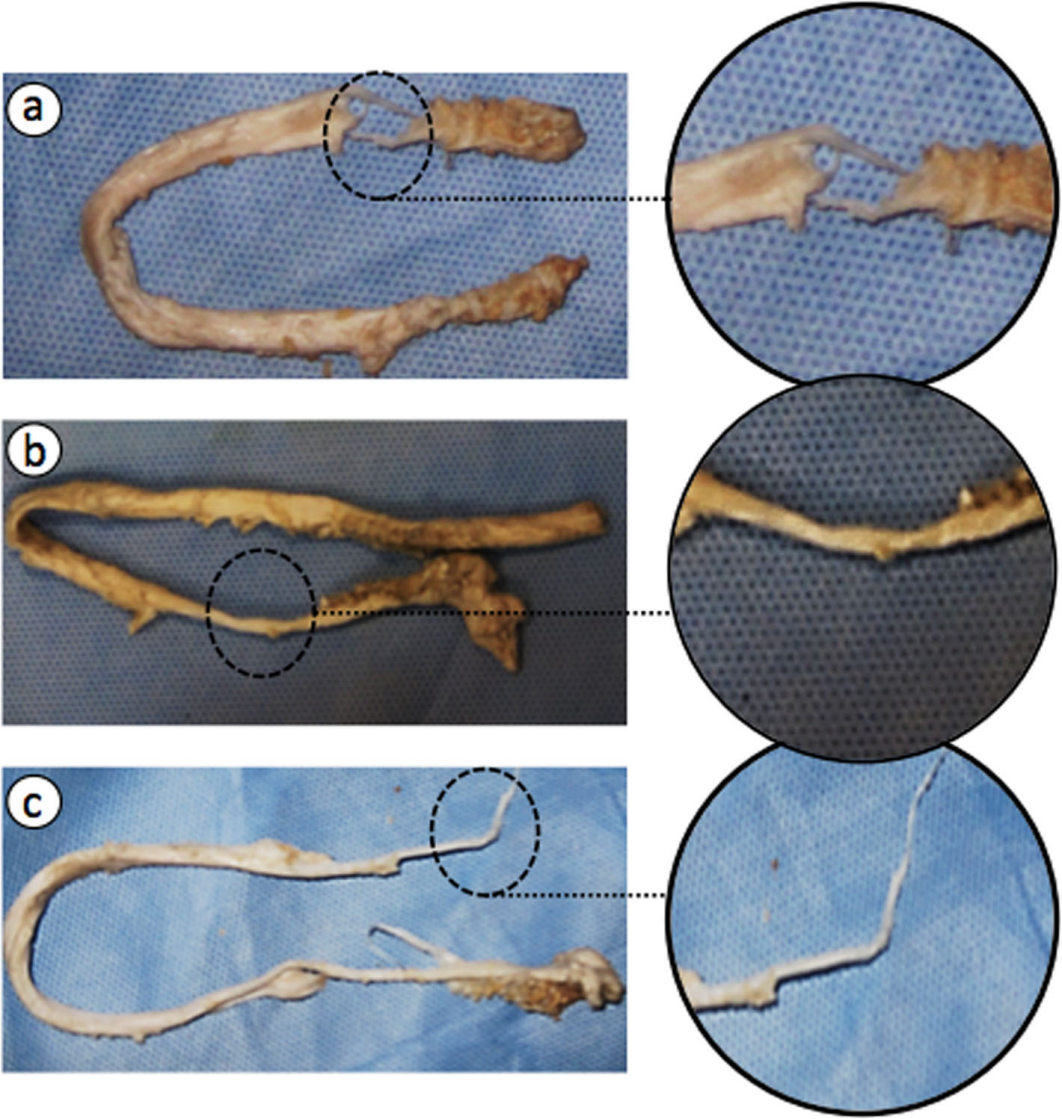
Three modes of the graft failure observed in this study: **a** Transverse rupture of graft’s fibers near to loading site (region A, Fig. 1); **b** Necking of the middle section of the graft material; and **c** Necking of the graft in region A (Fig. 1)

In loading grafts to failure (step c), even though no significant difference was observed between maximum loads at failure, and the stiffness of graft-bone-screw construct, between HSTIS and LSTIS groups (Table 1), but a noticeable difference between total graft displacements at failure was evident, between the two groups, i.e. 9.62 ± 1.42 mm for LSTIS group, compared with 7.31 ± 1.14 mm, for HSTIS group (*P* < 0.1) (Table 1). Moreover, the proximal site of fixation, near to loading site, i.e. region A, Fig. 1, was found to be the weakest section in all constructs. Different forms of graft failure were observed, i.e. transverse detachment of grafts’ fibers (Fig. 5a), necking of the middle section of the graft material (Fig. 5b), and necking of the graft in the insertion area (Fig. 5c). Another noticeable point is related to the major types of grafts’ failure in each group. The transverse cut of grafts’ fibers for LSTIS, i.e. 62.5 % of the samples of this group (5 out of 8), and necking of grafts in the HSTIS, i.e. 71.4 % of this group constructs (5 out of 7), were found to be major types of grafts’ failures. Necking in the middle region of the graft was another type of the graft failure, which was seen just in one sample, out of 7 samples, in HSTIS group.

**Table 1:** Summary of results in loading the graft up to failure: Stiffness of graft-bone-screw construct, Maximum load at failure and Total graft displacement in both groups with their corresponding *P* values

Measured parameter	LSTIS	HSTIS	*P* value
Stiffness of graft-bone-screw construct (N/mm)	107.9 ± 6.4	123.4 ± 27.7	*P* = 0.152
Maximum load at failure (N)	292.8 ± 163.4	360.2 ± 155.6	*P* = 0.415
Total graft displacement (mm)^a^	9.6 ± 1.42	7.3 ± 1.14	*P* = 0.086

^a^It is defined as the difference between initial position of graft after preconditioning step of loading and its position at failure point, along bone tunnel direction

## Discussion

There are still some concerns, regarding the ACL reconstruction using interference screws, such as the risk of early graft fixation failure, slippage, and laceration. Considering that an intact ACL experiences a gradual increase in stiffness as it gets closer to the point of insertion into the bone [29], it was hypothesized here that by increasing the slope of the interference screw and thus mimicking a natural ACL structure, the stability of fixation will increase. In order to check the validity of the hypothesis, two custom-made metallic interference screws were designed and fabricated, i.e. lower slope tapered interference screw (LSTIS), and higher slope tapered interference screw (HSTIS), and the performance of the fabricated screws were compared through experimental tests on graft-bone-interference screw constructs. The diameters of both screws in one-third of their length, from the tip of the screw, was equal to the bone tunnel diameter, which were gradually increased to 8.5 mm (in LSTIS), and 9.5 mm (in HSTIS), i.e. 1 and 2 mm greater than bone tunnel diameter (Fig. 1). Thus, in the LSTIS group, the average of screw diameter in one third of its length from tip (Fig. 1, region A) was larger, compared to the HSTIS group, but in the rest of its length (Fig. 1, regions B and C) was smaller.

To compare the capability of HSTIS and LSTIS in improving the initial stability of the reconstructed anterior cruciate ligament, stiffness of each graft-bone-screw construct was measured through applying a sub-failure incremental cyclic loading. Results of this work proved superiority of the HSTIS group, in terms of the graft-bone-screw stiffness, compared to the LSTIS group, especially in cyclic loading with the peak values of 100, 150 N (Fig. 4a). Stiffness of an intact femur-ACL-tibia complex of human cadaver knee, under approximately similar incremental cyclic loading protocol, was measured in Scheffler et al.‘ study [22]. Their reported mean stiffness of the samples’ constructs at cycles with peak values of 100, 200, and 300 N, were 43.8, 76.3, and 92.6 N/mm, respectively [22]. The mean stiffness values of the bone-graft-screw stiffness in the HSTIS group of this work, for the same cycles as the ones used in Scheffler et al.‘ study, were found to be 40.73, 68.99, and 102.24 N/mm. These values are closer to those of intact ACL in Scheffler et al.‘ study [22], compared with the LSTIS group, with the mean stiffness values of 27.82, 66.05, and 100.9 N/mm, respectively (Fig. 4a). Thus, it seems that fixation of the grafts with HSTIS better bio-mimick an intact ACL function, compared with LSTIS. Interesting to note that the values of stiffness for both constructs were in the same range, in higher loading levels, despite their different performance in terms of laxity and energy loss parameters (Fig. 4b and c). This observation can be explained by considering the definition of the construct’s stiffness, i.e. in each load level, stiffness represents reversible elongation of the graft in the fixation area, slippage of the graft from the bone tunnel, and graft fibers’ damage. In lower loading levels, the graft slippage and energy loss were predominate in LSTIS (Fig. 4b and c), and thus higher stiffness for HSTIS can be observed (Fig. 4a). However, due to a smaller average diameter of the HSTL in the region A of the fixation (Fig. 1), by increasing the load, the applied force may overcome squeezing pressure caused by interference screw, which causes more reversible elongation of the graft in HSTL group, compared with LSTIS group. Elongation of the graft leads to an increase in the working length of the graft, and by considering the fact that a longer tendon graft will undergo a larger elongation than a shorter graft and thus lower structural stiffness[22]. Therefore, it may can be explained why we observed almost the same stiffness of the construct for the two groups (Fig. 4a), for the higher loading levels, despite higher graft slippage in LSTIS group (Fig. 4b). Although elongation of the graft in the fixation site is not desirable, it is noteworthy that it does not mean the same performance of these two groups. These pieces of evidence suggested that HSTIS provides more flexibility in fixation site in higher load levels, which may indicate a more effective load transfer, and thus minimizes stress concentration in higher load levels, while LSTIS experiences graft damage and /or graft slippage (Fig. 4b an 4c), which is not reversible.

Graf laxity is an important concern associated with the ACL reconstruction when employing interference screws. The graft laxity can be caused by graft’s fiber damages, and/or graft slippage from the bone tunnel, without including the elongation of the tendon graft itself. The graft laxities found in this work for both groups were initiated by loads that were well below the failure load (see Fig. 4b) and showed an increase when the peak load was increased (see Fig. 4b). Moreover, mean graft laxity and energy loss measured for graft fixed with HSTIS was less than that of LSTIS for all loading cycles, especially at loading cycle with peak values of 100 and 150 N, in which the difference between LSTIS and HSTIS laxities were significant (*p* < 0.05) (Fig. 4b). This observation regarding the graft laxity and energy loss indicates that different body slopes of the screw will likely lead to a different performance of the reconstructed ACL in early stage of rehabilitation. Nonetheless, the results of this work showed that there is an insignificance difference between graft laxity and energy loss in two groups for the load greater than 150 N. These insignificant differences in graft laxity parameter, as well as energy loss can be due to the reduction of survived samples numbers in cycles with higher peak values, especially in LSTIS group. As was mentioned in the result section, three (out of eight) subjects were failed before reaching the peak load value of 200 N in sub-failure loading phase, which can directly affect the statistical analysis’s results.

By comparing the graft laxity parameter in the HSTIS group (Fig. 4.b) with those reported in Scheffler et al.’ study [22], using a similar protocol of loading, one may hold promise for superior behavior of the HSTIS to none-tapered metal interference screws. In their study [22], the graft laxity was measured in the case of fixing by Smith & Nephew RCI interference screw, with a diameter of 7 mm and length of 25 mm in the bone tunnels, with diameters ranging from 8 to 9 mm [22]. The graft laxity, in load cycle with a peak value of 200 N, was reported to be 3.0 ± 3.8 mm [22], which is greater than the corresponding value for both HSTIS and LSTIS groups of the current study, which were 1.16 ± 0.56 mm 2.49 ± 1.00, respectively. Furthermore, in Micucci et al.’ study [3], graft laxities, in the case of fixation by screws with diameters equal to 1 mm smaller, 1 mm and 2 mm greater than, the bone tunnel, were measured with a video analysis technique, and with photo-reflective markers, while the graft was experiencing a cyclic loading, ranging from 50 to 250 N at the frequency of 2 Hz, for a total of 1,500 cycles. The least graft slippage in their study was reported to be 2.65 ± 2.38 mm, for the screw with a diameter equals to the bone tunnel [3], which is greater than the graft laxity measured for HSTIS in this study, in cycle with a peak value of 250 N, i.e. 2.54 ± 1.02 mm. However, Miccuci et al.’ results have been reported after applying 1500 cycles of loading, and the reported values for 100 cycles of loads in their study are less than the corresponding values for both HSTIS and LSTIS groups. As a result, preponderance of the HSTIS to none-tapered bio-interface screws cannot be claimed in this study, and it seems that manufacturing the HSTIS with biodegradable materials can be deemed as a good option for improving its mechanical behavior.

Another cause of graft fixation failure in ACL reconstruction surgery through using interference screws, which can be observed clinically, is the graft laceration. In this study, in order to investigate the effect of the body slope of the screws on the graft damage, graft mode of failure for each sample was recorded. It was found that the graft and screw engagement in region A was the weakest site in all constructs (Fig. 1). However, the mode of graft failure in the HSTIS group was mostly necking of the grafts, and the samples did not fail due to screw threads cuts, which was mostly the mode of failure in the LSTIS group (see Fig. 5a). In previous studies, type of graft failure was only determined in terms of graft slippage, deterioration of graft material or failure at the mid-substance of the graft [34, 35]. Thus, due to the paucity of the data in the current literature on the subject of grafts’ fibers damages, comparison with previous work is not possible here.

Based on the evidence provided in terms of lower stiffness, greater graft’s fibers damages, higher graft laxity, and displacement in the LSTIS group, compared with the HSTIS group (Table 1; Figs. 4 and 5), it can be speculated that in the ACL reconstruction surgery, through using a tapered interference screw, the risk of early graft fixation failure can be minimized through controlling the pressure and contact area of the screw and graft, by means of precisely determining body slope for the screw. It seems that in regions B and C (Fig. 1), the smaller average diameter of the screw and lower body slope of the LSTIS cause less friction, compared to the HSTIS, which consequently lead to a greater displacement of the graft in the former group. Subsequently, the slippage of the graft in regions B and C will be transmitted to region A, near to the loading exertion point, due to the direction of the applied load, i.e. pulling the graft outside of the bone tunnel (Fig. 2). Finally, it can be deemed that this transmitted graft to region A, in the LSTIS group, due to a larger mean diameter of screw (Fig. 1), compared to the HSTIS, will be exposed to higher average contact pressure, which leads to a transverse cut of the graft’s fibers (see Fig. 5). On the other hand, higher body slop of HSTIS group, in conjunction with a smaller mean diameter of the HSTIS in region A, compared to the LSTIS, prevents transverse cut of the graft at the most vulnerable regions of the fixation (Fig. 5). Furthermore, loading behavior of the HSTIS, in higher loading levels, as was already discussed, showed that the HSTIS provides more flexibility in the fixation site, reflecting a more effective load transfer, and thus minimizes stress concentration, and that is why necking of the graft, instead of transverse cutting, can be observed in this group (Fig. 5c). Therefore, it can be suggested that in regions B and C, major slippage of the graft takes place, which could be transferred to hazardous region A that can cause further damages on graft fibers, thus an adequate contact pressure must be applied in regions B and C, while high contact pressures should be avoided in region A.

The following points should be taken into consideration while one is trying to interpret the results of this work. First, *in-vitro* tests’ results can give us information about the initial stability, but they are unable to evaluate the mechanical behavior of the bone-graft-interference screw construct after graft healing and remodeling processes, which can alter graft tissue’s mechanical properties [36]. Secondly, stress distribution within the graft and on bone tunnel can have an influence on bone tunnel widening during the healing and remodeling processes and consequently can affect graft fixation stability, which was not taken into account in this study. Thirdly, it should be noted that extensor-digitrom of bovine [37, 38], instead of human hamstring tendon, was used in this investigation. Fourthly, synthetic bone, similar to a dense cancellous bone, was used here, in order to avoid cadaver’s wide range variation in BMDs, as well as non-homogeneity of real bone, and thus make the comparison between the LSTIS and HSTIS more logical. Lastly, even though the ASTM standards, i.e. F543-07 and F2502, have been used to evaluate the performance of the interference screws, but these standard tests mainly focused on strength of the interference screws’ fixation in the bone, without considering the graft in the bone tunnel [39]. However, to replicate the real biomechanical situation and evaluate current ACL reconstruction problems, i.e. graft slippage, irritation, and laceration, a full ACL reconstruction structure (the graft, bone and screw) was used in this study, similar to some previous biomechanical investigations (e.g. [22 and 23]). Furthermore, we did not discriminate between the structure’ components (the graft, bone and screw), in this work. Since the same bone and tendon specimens were carefully chosen, so assuming the same material and structural properties for the hard and soft tissues should seem logical, and thus comparing the biomechanical test results in two groups should be only the reflection of interference screws’ designs.

## Conclusions

In the ACL reconstruction surgery, by using the bio-mimicked tapered interference screw, the risk of early graft fixation failure can be reduced through controlling the contact area of screw and graft, and thus by adjusting pressure distribution at the screw-graft and graft-bone interfaces, by means of screw body slope, and consequently by adjusting the screw-bone tunnel diameters ratio gradient along the interference screw. Based on this study, it can be concluded that in the area near the load exertion site, region A (Fig. 1), engagement of screw and graft can cause graft damages (Fig. 5), and thus high contact pressure should be avoided in that region. Moreover, major slippage of graft occurs in regions B and C (Fig. 1), which might migrate to the critical region A, and cause further damages, thus a proper graft fitting must be maintained in regions B and C. By increasing the diameter of the interference screw linearly, similar to the custom-made HSTIS of this study, with a greater body slope than that of LSTIS, a greater screw-bone tunnel diameter ratio gradient along the screw can be gained, and thus it can better bio-mimic the intact ACL attachment behavior. Since just two most probable efficient body slopes were studied here, in order to discover new aspects of the effects of body slope of the interference screw and answering the key question of “what is the optimal slope for an interference screw that can result in the most favorite outcome of ACL reconstruction surgery?’’ further attention and investigations need to be made in the future.

## Data Availability

The datasets used during the current study are available from the corresponding author on reasonable request.

## Abbreviations

ACL: Anterior cruciate ligament.
LSTIS: Lower slope tapered interference screw.
HSTIS: Higher slope tapered interference screw.
